# Fish reproductive phenology shifts with increasing temperature and year

**DOI:** 10.1098/rsbl.2024.0240

**Published:** 2025-01-08

**Authors:** S. T. Koenigbauer, M. L. Cubbage, L. D. Warren, J. M. Tellier, O. M. Selz, G. G. Sass, T. O. Höök

**Affiliations:** ^1^ Department of Forestry and Natural Resources, Purdue University, Forestry Building, 195 Marsteller Street, West Lafayette, IN 47907, USA; ^2^ Indiana Department of Environmental Management, 2525 Shadeland Avenue, Indianapolis, IN 46219, USA; ^3^ Michigan Department of Environment, Great Lakes, and Energy, 525 W. Allegan Street, Lansing, MI 48933, USA; ^4^ Federal Office for the Environment (FOEN), Aquatic Restoration and Fisheries Section, Bern 3011, Switzerland; ^5^ Wisconsin Department of Natural Resources, Office of Applied Science, Escanaba Lake Research Station, 3110 Trout Lake Station Drive, Boulder Junction, WI 54512, USA; ^6^ Illinois-Indiana Sea Grant College Program, Purdue University, Forestry Building, 195 Marsteller Street, West Lafayette, IN 47907, USA

**Keywords:** climate change, meta-analysis, migration, spawning, temperate, warming

## Abstract

Temperate fishes often spawn in response to environmental cues, such as temperature, thereby facilitating larval emergence concurrent with suitable biotic and abiotic conditions, such as plankton blooms. Climatic changes may alter the reproductive phenology of spring- and autumn-spawning freshwater fish populations. Such effects may depend on the sensitivity of reproductive phenology to ambient temperatures. We applied a meta-analysis approach to test whether annual temperature and year affected fish reproductive phenology. Based on preliminary tests in walleye (*Sander vitreus*) and Lake Constance whitefish (*Coregonus arenicolus*), we hypothesized that increasing temperature would promote earlier spring-spawning and later autumn-spawning. We found spawning was significantly earlier in the spring and later in the autumn. We found that migration of autumn-spawning species occurred earlier with warmer temperatures, implying that with increasing temperatures, migrating autumn-spawning species will increase residence time in tributaries. We also found that spring-spawning fishes reproduced earlier in more recent years, while we observed no significant effect in autumn-spawners. Spring- and autumn-spawning fishes displayed interannual variation in spawning dates (mean range of 34.4 and 27.0 days over 33.9 years, respectively), with spring-spawning fishes displaying a significantly broader range in spawning dates.

## 1. Introduction

Temperate freshwater fishes display seasonal reproduction, generally spawning during spring with larval emergence and growth before winter or spawning during autumn with overwintering egg incubation and spring larval emergence. Although seasonal spawning is generalizable among temperate freshwater fishes, populations and individuals within species display interannual variability in reproductive timing, or phenology. Previous research has shown that reproductive phenology is partially driven by environmental variables such as flow regime, photoperiod and temperature [1–4]. Photoperiod is relatively consistent across years and may potentially restrict variation in fish reproductive phenology, tempering the influence of other environmental cues. The influence of temperature on reproductive phenology is relatively well studied for autumn- and spring-spawning freshwater and anadromous fishes [5–11]. However, the generality of thermal effects on freshwater fish reproductive phenology and the magnitude of variation in reproductive phenology across populations could be better synthesized through systemic analysis of previous studies.

The reproductive phenology of fish populations has presumably evolved in part to facilitate offspring emergence during times favouring successful growth and survival. Slight shifts in the timing of larval fish emergence can have profound effects on early life survival and subsequent recruitment [12–15]. Long-term shifts in reproductive phenology and emergence could lead to decreased recruitment success. Alternatively, if larval fish emergence shifts similarly to environmental conditions (e.g. temporally variable prey availability), fish populations may maintain relatively strong recruitment [16].

**Figure 1.**
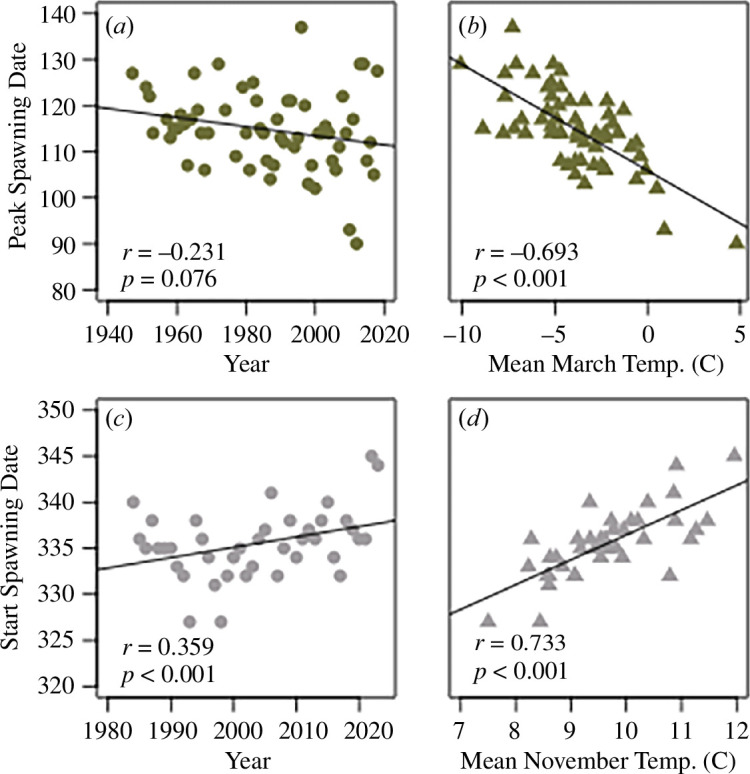
Scatterplots of peak spawning dates for walleye (*Sander vitreus*) (*a,b*) and initial spawning dates for European whitefish (*c,d*) by year and temperature. Each point represents a single spawning season. Walleye were surveyed in Escanaba Lake, United States by the Wisconsin Department of Natural Resources, and the European whitefish (*Coregonus arenicolus* and *Coregonus macrophthalmus*) were surveyed during spawning fisheries in Lower Lake Constance by the joint efforts of the fisheries authorities of Germany and Switzerland. Walleye are a spring-spawning species, and the two European whitefish are an autumn-spawning species.

Climate change has and will continue to alter temperatures experienced by temperate freshwater fishes, potentially affecting reproductive phenology. Combined land and ocean temperature has increased at an average rate of 0.06°C per decade since 1880, and the 10 warmest years on record globally occurred after 2005 [17]. As potential examples, consider analyses of annual temperature and year effects on peak reproductive dates in spring-spawning walleye (*Sander vitreus*) from Escanaba Lake and the start of spawning season in autumn-spawning European whitefish (*Coregonus arenicolus* and *Coregonus macrophthalmus*) from Lower Lake Constance (figure 1; see electronic supplemental materials for details). These populations demonstrate the general expectations that accelerated warming in spring could trigger earlier fish spawning in the spring, and persistent warm temperatures could delay fish spawning in the autumn. As demonstrated for walleye, such shifting reproductive phenology may affect population genetic variation and recruitment [18,19].

To anticipate whether populations may respond to past and future environmental changes, we evaluated potential shifts in reproductive phenology with increasing temperature and year in temperate freshwater fishes. Specifically, we focused on indices of spawning dates of fish populations (and not earlier stages of gonadal development). We assessed the consistency of temperature and year effects on reproductive phenology by synthesizing results from studies of different systems, species and time periods. Our objectives were to (i) test for the effect of temperature and year on reproductive phenology for autumn- and spring-spawning species and (ii) test for the potential magnitude of shifts in reproductive phenology over time. Given that many autumn-spawning temperate freshwater fishes undertake a migration several months before spawning, we considered variation in both timing of migration and reproduction. In addition, we examined whether increasing temperature had a stronger effect on spawning date in different system types (lotic and lentic). We hypothesized that warmer temperatures would lead to earlier reproduction for spring-spawning species and later reproduction for autumn-spawning species. We also hypothesized that the magnitude of interannual variation in reproductive phenology would not vary between spring- and autumn-spawning fishes.

## 2. Methods

### (a) Meta-analysis

To test for broad effects of temperature and year on reproductive phenology, we used a meta-analysis approach. We selected a query that yielded a relatively large number of papers: ‘Topic Search = (spawn* or reproduc* AND temperature AND fish AND timing AND freshwater)’ in ISI Web of Science on 7 April 2020 and examined all resulting papers for relationships between temperature and measurements of reproductive timing in wild freshwater or anadromous fish populations. We then performed backward and forward analyses of papers that met study criteria from the initial search. Backward searches entailed searching through all references in a selected study, and forward searches entailed examining all papers that cited a selected study. To update our analysis, we supplemented our meta-analysis with additional papers from the U.S. Geological Survey Fish and Climate Change Database (FiCli), which can be accessed at (https://www.usgs.gov/tools/fish-and-climate-change-database-ficli; [20]). We accessed the database on 15 February 2024 and reviewed all papers for the same criteria as our Web of Science search. In the FiCli database, we filtered the response category column by ‘phenological’ and assessed all resulting rows.

For our analysis, we selected studies that reported (i) annual reproductive or migration date and (ii) a corresponding annual temperature index for a minimum of 4 years. We only included studies of fish populations that spawned in the autumn or spring, as indicated by the study authors. To synthesize across studies that varied in design, we collected effect sizes in the form of correlation coefficients that estimated an effect of temperature on reproductive timing from each study. Measures of annual reproductive timing were most commonly based on median capture (incidence of ripe females in sampling gear) or observation (indirect detection such as fish counters or telemetry) of mature fish on spawning grounds but also included observations of the onset of spawning through detection of adults engaging in spawning activities (e.g. nest-building or egg deposition). Temperature was most often measured as mean water temperature during spawning (e.g. mean water temperature of a month or season), but a smaller proportion of studies considered mean annual water temperatures, mean air temperatures at monthly, seasonal or annual scales, the date when a particular temperature (e.g. established temperature for spawning cue) was reached in a given year or the date of ice-out. Plausibly, annual temperatures could affect timing of initial spawning or peak spawning by a population, and different reproductive indices differentially measure these effects (we prioritized peak spawning when available). Although fish spawning is presumably most affected by water temperature, when water temperature was unavailable, we included studies that measured air temperature (assuming correlation between air and water temperature). When estimating mean spawning date, studies that reported growing degree days did not consistently index the same time periods, limiting their utility as predictors for reproductive timing. Many studies directly reported relevant correlation coefficients between annual temperature indices and reproductive timing, and for other studies, we estimated correlation coefficients using image analysis (ImageJ v. 1.53j May 2021). Our literature search methods resulted in a systematic sample of relevant studies. Inevitably, there were studies not detected in our search. Consistent with accepted meta-analysis methods, we suggest it is more appropriate to systematically sample studies in a replicable and relatively unbiased manner than to select studies in an *ad hoc* manner.

Many studies reported multiple correlation coefficients relating reproductive phenology to an annual temperature index, such as reporting reproductive timing of multiple species or populations. We used two approaches to analyse these effects. (i) In the inclusive analyses, we did not include multiple effects relating to the same fish observations (e.g. the effect of air temperature and water temperature on mean spawning date). (ii) In the conservative analyses, to address potential dependency of multiple species-specific effects from the same publication, we further restricted to only include a single, randomly selected effect for each species reported in a publication. For example, for a study that allowed for calculation of effect sizes for two species in five river systems, the inclusive analysis would include all 10 effects, and the conservative analysis would include one effect for each species.

After collecting effects, we transformed correlation coefficients (*r*) to Fisher’s *Z* equation (2.1), which more closely approximates a normal distribution [19] and calculated associated sampling variance (var(*Z*); equation (2.2)). Because study variables differed, we assigned negative signs when reproductive timing was earlier with increasing temperature or year, and positive signs when reproductive timing was delayed.


(2.1) $$Z_{i}\;=\;\frac{1}{2}\left[In\left(1+r_{i}\right)\;-\;In\left(1-r_{i}\right)\right]$$

(2.2) $$Var\;\left(Z_{i}\right)\;=\;\frac{1}{n_{i}-3}$$

We calculated a weighting factor (*W_i_*) for each effect size as its inverse variance [21] and weighted mean effects for each season group equation (2.3).


(2.3) $$\overset{-}{Z}\;=\;\frac{\sum Z_{i}W_{i}}{\sum W_{i}}$$

We applied parametric statistics to our effect sizes and considered overall relationships as significant if mean effect size and corresponding 95% CIs excluded zero. In addition to estimating mean effect sizes for each spawning season, we categorized effects by lentic and lotic systems and estimated mean effects for each system type in the spring and autumn.

### (b) Year effect on reproductive phenology

Our primary focus was to test the effect of annual thermal conditions on reproductive phenology. Considering increases in global temperature over the past several decades, a related question was whether annual reproductive phenology has trended over time. As an *a posteriori* analysis, we considered temporal shifts in reproductive phenology for the three study groups (spring reproduction, autumn reproduction and autumn migration). To this end, we evaluated the papers from the reproductive phenology–temperature analysis for effects of year on reproductive phenology. We obtained correlation coefficients from studies relating year and annual timing of reproduction. We applied similar meta-analysis methods as described in equations (2.1)–(2.3) and for both inclusive and conservative datasets.

### (c) Magnitude of reproductive phenological variability

To assess plasticity of reproductive phenology, we analysed the range of reproductive timing across years. For each effect size calculated for the inclusive analysis, we recorded the range of dates when reproduction occurred across years (i.e. the latest day of year minus the earliest day of year of annual reproductive timing over all years sampled), and the number of years data was collected. Studies that did not provide reproductive dates were excluded. We hypothesized that the range of reproductive dates would increase with the number of years sampled. To compare the magnitude of change in reproductive phenology among spring-spawning populations and autumn-spawning populations, we used R package ‘emmeans’ to conduct analysis of covariance (ANCOVA) with reproductive range as the response variable, reproductive season as the explanatory variable and number of years sampled as a covariate [22]. Initially, we included an interaction term between the number of years in a study and the study season, but this term was insignificant (*p* > 0.05), and we excluded it from the final model. We excluded migration from this analysis considering it is a separate process from reproductive phenology. Statistical significance was determined at the *α* = 0.05 level.

### (d) Assessing publication bias

Pressures on researchers and journals to publish significant, directional effects can contribute to publication bias. Further, researchers may be more inclined to report patterns in species where shifts in reproductive phenology are more prominent. For example, many published studies of reproductive phenology in autumn-spawning fishes focused on salmonids (see electronic supplemental materials for taxonomic distribution of effects in our analyses). In part due to such biases, individual effects may not be evenly centred on an overall mean effect and may confound interpretation of overall effect sizes. To test for publication bias, we applied Egger’s tests of asymmetry for each spawning season group based on the inclusive datasets [23]. We used the ‘metafor’ package in R to conduct these Egger’s tests [24].

## 3. Results

### (a) Literature search

The Web of Science search yielded 860 publications. Through the initial paper review and subsequent forward and backward analyses, 39 papers with a total of 76 effects of temperature and 102 effects of year on reproductive phenology were included. Through the supplemental search of FiCli, 340 papers were reviewed and an additional 13 papers were retained that were not found in the Web of Science search, with a total six effects of temperature and 43 effects of year on reproductive phenology. For effects of temperature on reproductive phenology, 28 effects for spring-spawners, eight for autumn-spawners and 46 effects of temperature on autumn migration timing were found. For effects of year on reproductive phenology, 52 effects for spring-spawners, 14 for autumn-spawners and 79 effects of year on autumn migration timing were found. The meta-analysis search yielded results from seven taxonomic orders (electronic supplementary material, tables S1 and S2). The most common orders observed were Perciformes in spring-spawning species and Salmoniformes in autumn-spawning species. Most effects included in our analyses were extracted from studies in North America, but Asia, Australia and Europe were also represented (electronic supplementary material, tables S1 and S2). References used in our meta-analyses can be found in electronic supplemental material. Data and code used for our meta-analyses can be found in the Purdue University Research Repository (https://doi.org/10.4231/KT69-1R91; [25]).

### (b) Temperature effect on reproductive phenology

**Figure 2.**
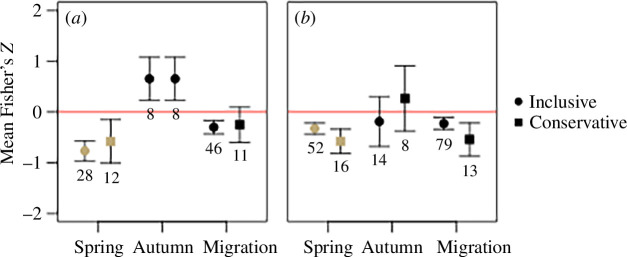
Meta-analysis results for effects of temperature (*a*) and year (*b*) on fish spawning phenology. Points are mean Fisher’s *Z* effects weighted by inverse study variance, and bars represent 95% CIs. If confidence intervals did not cross 0, a significant effect was concluded. Positive effects implied later spawning, and negative effects imply earlier spawning. Numbers below denote the number of effects used to calculate a mean. Studies were grouped by species that spawn in the spring and autumn, with an additional group for studies of autumn migration (not exact spawning dates). Inclusive means include all available effects, while conservative means include only one effect per paper.

For spring-spawning fishes, increasing temperatures led to significantly earlier reproductive timing based on both the inclusive ($\overset{-}{Z}$ = −0.767 ± 0.199) and conservative analyses ($\overset{-}{Z}$ = −0.579 ± 0.431; figure 2). Of the 28 effects from spring-spawning fishes, 16 were from lentic systems ($\overset{-}{Z}$ = −0.849 ± 0.322), and 12 were from lotic systems ($\overset{-}{Z}$ = −0.679 ± 0.205). For autumn-spawning fishes, increasing temperatures led to significantly later spawning in the autumn ($\overset{-}{Z}$ = 0.653 ± 0.424; figure 2*a*). Because there were no studies that reported multiple effects, the inclusive and conservative analyses were identical. Of the eight effects from autumn-spawning fishes, four were from lentic systems ($\overset{-}{Z}$ = 1.235 ± 0.144) and four were from lotic systems ($\overset{-}{Z}$ = 0.431 ± 0.487). Autumn-spawning fishes migrated significantly earlier with warmer temperatures in the inclusive analysis ($\overset{-}{Z}$ = −0.301 ± 0.133) but not the conservative analysis ($\overset{-}{Z}$ = −0.253 ± 0.349; figure 2*a*).

### (c) Year effect on reproductive phenology

For spring-spawning fishes, timing of reproduction occurred progressively and significantly earlier (years included: 1938–2012) based on both the inclusive (*Z* = −0.326 ± 0.111) and conservative analyses (*Z* = −0.577 ± 0.238; figure 2*b*). For autumn-spawning fishes, the direction of mean weighted effect of year on reproduction varied between the inclusive and conservative analysis (years included: 1890–2010). A negative weighted mean effect of year on reproductive phenology was observed when including all effects ($\overset{-}{Z}$ = −0.189 ± 0.489), and a positive weighted mean effect was observed using the conservative analysis ($\overset{-}{Z}$ = 0.266 ± 0.642; figure 2*b*), but neither were statistically significant. The timing of autumn migration occurred progressively and significantly earlier over the past century (years included: 1933–2012) based on the inclusive ($\overset{-}{Z}$ = −0.229 ± 0.117) and conservative analyses ($\overset{-}{Z}$ = −0.541 ± 0.326; figure 2*b*).

### (d) Magnitude of reproductive phenological variability

**Figure 3.**
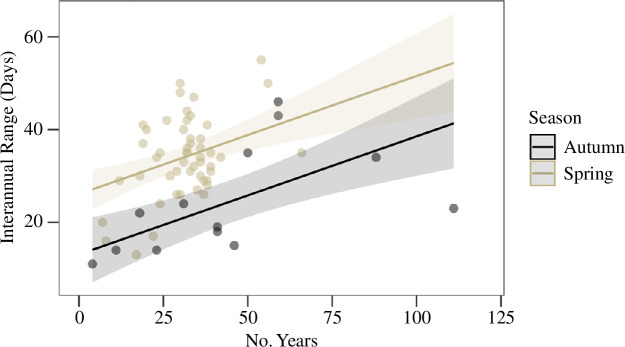
Interannual range of spawning dates from studies included in our meta-analysis according to study duration (i.e. number of years where spawning was observed). Each point represents an interannual time series of spawning dates. Trendlines and 95% CIs were estimated with an ANCOVA model. Accounting for slopes, studies of spring-spawners found more variation in spawning dates than in autumn-spawners.

For spring-spawning fishes, population-specific range of reproductive dates varied from 13 to 55 days (study durations from 7 to 66 years). For autumn-spawning fishes, the range of reproductive dates varied from 11 to 77 days (study durations from 4 to 111 years). One date range observation exceeded 1.5 times the interquartile range of all observations and was removed. Using an ANCOVA model, we first found an insignificant season–study duration interaction coefficient. After removing the interaction, both study duration (*p* < 0.001) and spawning season (*p* < 0.001) significantly predicted date range, and the overall model was also significant (*R*^2^ = 0.306, *F*_2,60_ = 14.67, *p* < 0.001). Using estimated marginal means, spring-spawning fishes had a significantly broader range of spawning dates on average (34.8 ± 2.4 days) than autumn-spawning fishes (21.8 ± 4.8 days) at the global mean study duration (34.2 years; figure 3).

### (e) Assessing publication bias

When assessing temperature–reproductive phenology based on the inclusive approach, the null hypothesis of symmetrical distribution of effects sizes for autumn-spawning fishes (z = 1.342, *p* = 0.180) or autumn migration (z = −0.605, *p* = 0.546) was not rejected, but there was significant evidence of asymmetry in spring-spawning fishes (z = −2.01, *p* = 0.045; electronic supplementary material, figure S1). For the conservative approach, no significant evidence of asymmetry in temperature-reproductive phenology effects for spring-spawners (z = −1.651, *p* = 0.099), autumn-spawners (same as inclusive dataset) or autumn migration (z = 0.547, *p* = 0.585) was observed. The effect sizes relating year and reproductive phenology under the inclusive approach were not significantly skewed for spring- (z = −0.267, *p* = 0.789) and autumn-spawning (z = 1.215, *p* = 0.224) fishes, but for autumn migration timing there was potential publication bias among effects of year on reproductive phenology (z = 4.700, *p* < 0.001; electronic supplementary material, figure S2). For the conservative approach, no significant evidence of asymmetry in year–reproductive phenology effects for spring-spawners (z = 1.291, *p* = 0.197), autumn-spawners (z = 0.598, *p* = 0.545) or autumn migration (z = 1.580, *p* = 0.114) was observed.

## 4. Discussion

Spring-spawning fishes reproduced earlier during warmer and more recent years, and generally displayed greater variation in reproductive phenology than autumn-spawning fishes. While we could not estimate a rate of phenological shift with increasing temperature in our dataset, Pauly & Liang (2022) reported a sinusoidal model that predicts fish will spawn 11.7 days earlier per 1°C increase in spring temperatures [26]. These shifts in reproductive phenology will likely result in altered hatch timing of larvae, especially as egg durations are relatively short among spring-spawners [26,27]. Concurrent larval emergence and spring plankton blooms is important for larval foraging, growth and survival [18,28], often coinciding with a critical period that contributes greatly to recruitment success [29]. Plasticity in reproductive phenology does not ensure that larvae experience ideal environmental conditions. For example, deviation from average peak spawning dates can coincide with weak age-0 walleye recruitment [18]. Potential mismatches between larval emergence and prey likely depend on the phenological shift of other trophic groups in response to increasing temperatures. In general, peak phytoplankton and zooplankton blooms have generally shifted earlier in recent decades with warming temperatures [30,31], yet individual zooplankton taxa vary in their response [32–34]. With projected future rise in global temperatures, spring-spawning fishes may continue to spawn progressively earlier, resulting in earlier emergence of larvae which will likely experience altered plankton assemblages in lentic systems or benthic macroinvertebrates in lotic systems. Additionally, phenological shifts in spawning and habitat utilization of competitor and predator species will likely further alter the realized stability of a population through climate change.

Autumn-spawning fishes reproduced later with warmer autumn temperature, which could influence egg stage duration. Egg stage durations of autumn-spawning fishes are typically longer than spring-spawning fishes, lasting up to several months [35–37]. For autumn-spawning fishes, increased warming rates can accelerate embryo development and contribute to earlier hatch time [38,39]. Many studies have documented changes in physiology (e.g. variation in larval length and a reduced yolk sac at hatch) and behaviour (e.g. larval swimming and explorative activity or outmigration tendency later in life) with increased incubation temperature that may affect different life-history traits [37–42]. However, these studies focused on incubation temperature, without including reproductive phenology as a factor. Thermally induced increased development rate may result in shortened incubation. Delayed reproductive timing may offset such reduced incubation time, and thus it is not manifest that larval emergence will occur earlier. Additional research into the combinations of delayed reproductive timing and increased spring warming rates should be considered to test for consequences for emergence timing and potential physiological and survival effects. Surprisingly, we found no significant trend of year on reproductive phenology in autumn-spawners. Potentially, other factors affecting autumn reproductive phenology, such as precipitation or photoperiod, confounded the relationship between year and mean spawning date in the populations in our analyses. Generally, we found fewer studies of autumn reproductive phenology in our search, and more focus on autumn-spawners would be insightful [43].

The juxtaposition of earlier migration and delayed spawning among autumn-spawning fishes could result in increased residence time in spawning habitats, such as rivers for anadromous species [44]. Increased residence time in spawning habitats could have consequences for migratory fish populations such as increased vulnerability to thermal stress [45] and terrestrial predators. Increasing fish residence time also has consequences for the ecosystems they inhabit, as migrations of autumn-spawning fishes can temporarily affect dissolved oxygen concentrations [46], primary production [47] and nutrient concentrations [48] in their reproductive habitats. Studies included in our analysis typically only recorded migration timing or spawn timing, leading to our conclusions of earlier migration and delayed spawning being derived from different populations and species. To further demonstrate potentially increased residence time among autumn-spawning populations, future research could incorporate migration and spawning phenology to test for consequences of such phenomena.

By applying a meta-analysis approach, we incorporated a diverse sample of studies that featured a variety of measurements associating variation in temperature and year with fish reproductive phenology. Meta-analyses are robust to alternate indices of temperature and spawning by evaluating unitless effect sizes. Beyond our examination of phenological correlation with temperature and year, we provided evidence of plasticity in reproductive phenology among temperate freshwater fishes, with reproductive timing within a study varying as much as 77 days among years. Controlling for study duration, spring-spawning fishes exhibited greater reproductive timing variation than autumn-spawning fishes. The reasons for this difference are unclear but may relate to various environmental cues differentially influencing reproductive phenology of these groups. For example, relatively consistent factors such as photoperiod may differentially affect [4,49] or limit future shifts in reproductive phenology. Although freshwater fishes display plastic reproductive phenology in response to thermal conditions, there will likely be constraints to such responses.

## Data Availability

All data and code for analyses associated with this manuscript can be found with open access in the Purdue University Research Repository [25]. Electronic supplementary material is available online [50].
